# Supplementary material for: Disparate Effects of Two Clerodane Diterpenes of Giant Goldenrod (Solidago gigantea Ait.) on Bacillus spizizenii
Source: Int J Mol Sci. 2024 Jan 26;25(3):1531. doi: 10.3390/ijms25031531 (PMC10855248; doi:10.3390/ijms25031531)
Supplement: Supplementary file 1 [file ijms-25-01531-s001.zip › Figure S 1.pdf]

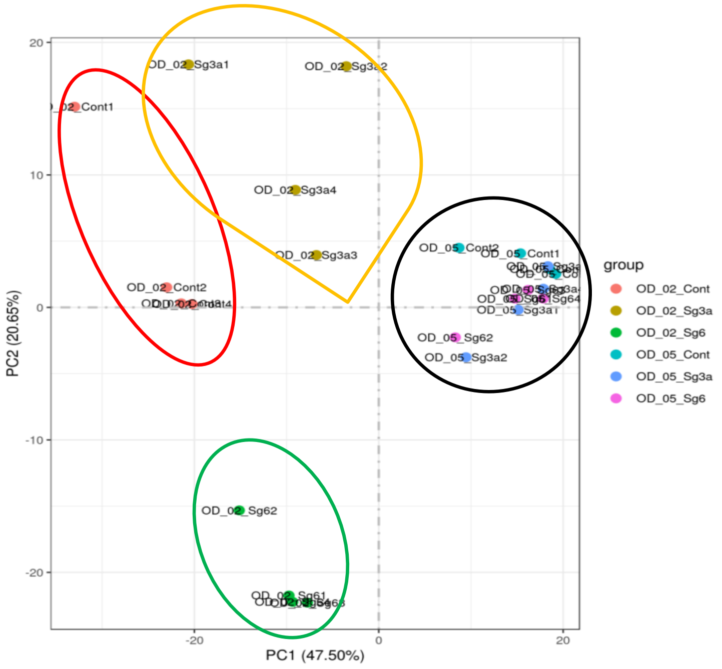

Figure S1. 2D principal component analysis of transcription results of samples obtained after diterpene (Sg3a and Sg6) treatments or in control (Cont) samples. *B. spizizenii* RNA was isolated from OD_{600}=0.2 samples after 5 hours, while RNA was purified from OD_{600}=0.5 samples after 1 hour incubation.
