# Supplementary material for: Disparate Effects of Two Clerodane Diterpenes of Giant Goldenrod (Solidago gigantea Ait.) on Bacillus spizizenii
Source: Int J Mol Sci. 2024 Jan 26;25(3):1531. doi: 10.3390/ijms25031531 (PMC10855248; doi:10.3390/ijms25031531)

Figure S4. Two-component systems gene activity changes of *B. spizizenii* after diterpene treatments.

Sg3a-5h vs. control-5h

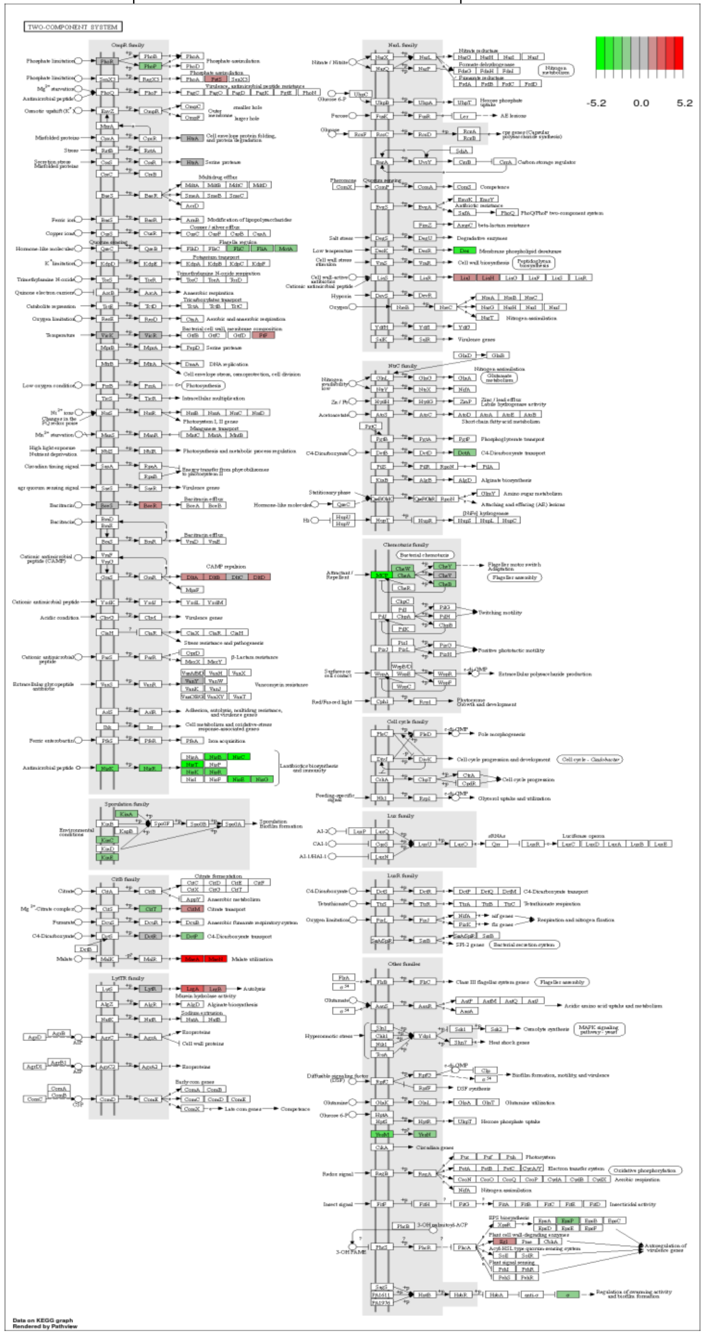

Sg6-5h vs. control-5h

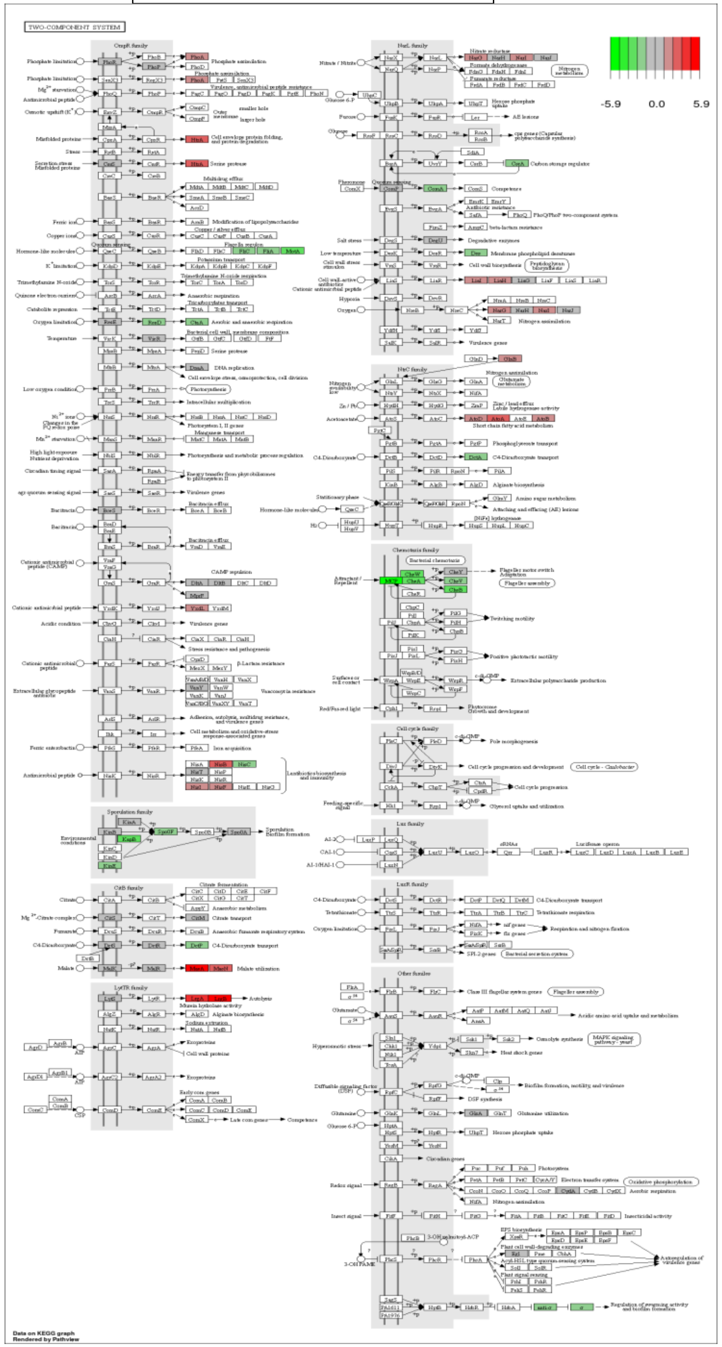

Sg3a-1h vs. control-h

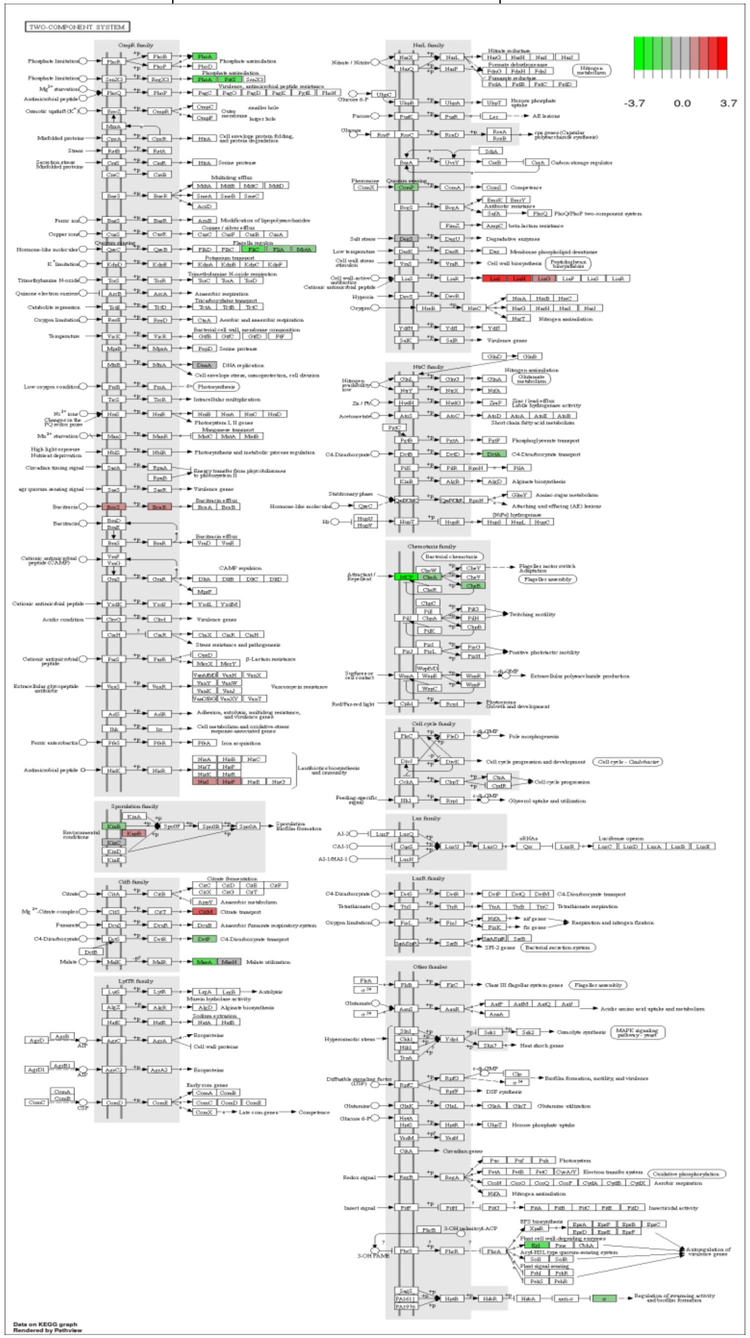

Sg6-1h vs. control-1h

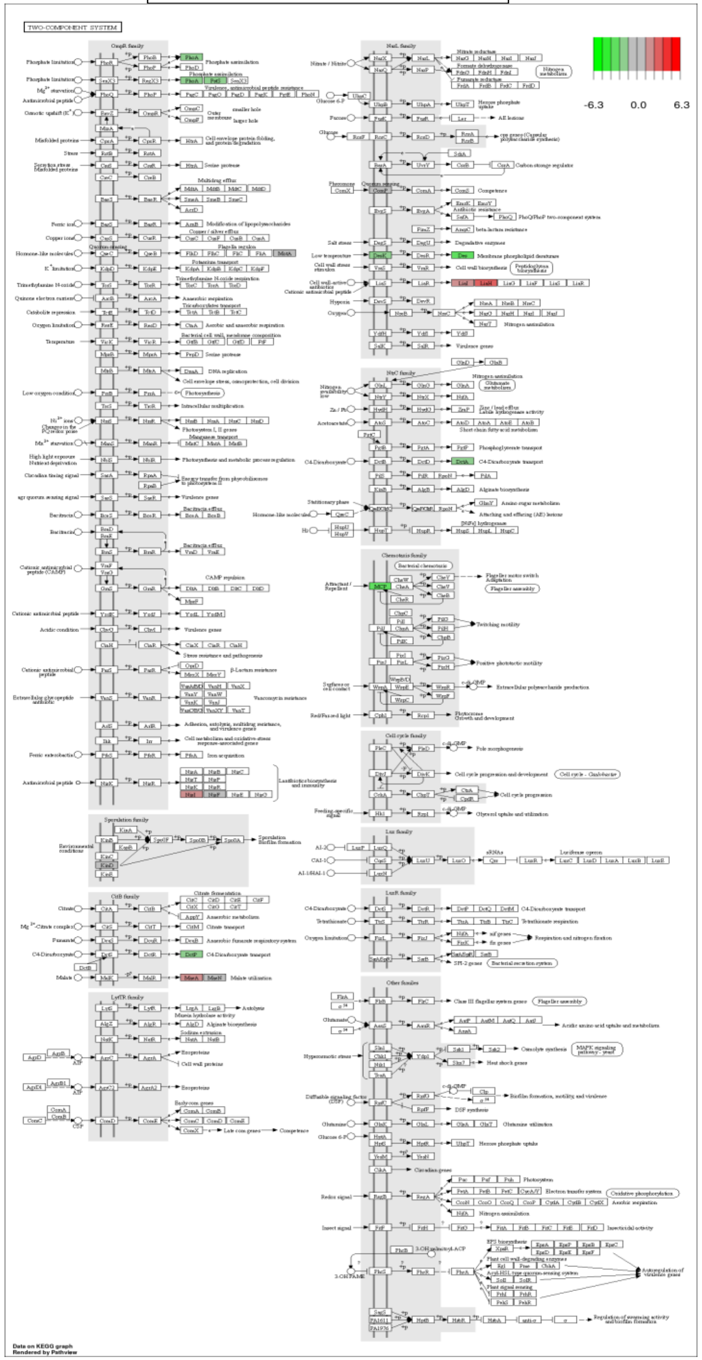

Supplement: Supplementary file 1 [file ijms-25-01531-s001.zip › Figure S4.pdf]
