# Supplementary material for: Disparate Effects of Two Clerodane Diterpenes of Giant Goldenrod (Solidago gigantea Ait.) on Bacillus spizizenii
Source: Int J Mol Sci. 2024 Jan 26;25(3):1531. doi: 10.3390/ijms25031531 (PMC10855248; doi:10.3390/ijms25031531)
Supplement: Supplementary file 1 [file ijms-25-01531-s001.zip › Table S2.pdf]

**Table S2. Overlapping *B. spizizenii* genes with different patterns that changed their activity after all types of treatments**

| gene_id | Sg3a-5h | Sg6-5h | Sg3a-1h | Sg6-1h | gene_description |
|---|---|---|---|---|---|
| gene:GYO_2763 | 1.2[a] | 1.6 | -2.0 | -2.1 | && PF00528:Binding-protein-dependent transport system inner membrane component, |
| gene:GYO_0471 | 1.2 | -1.7 | -1.3 | -1.9 | - && PF01674:Lipase (class 2) |
| gene:GYO_1034 | 1.5 | -2.3 | -1.1 | -1.3 | - && - |
| gene:GYO_4367 | 1.9 | -1.3 | -1.6 | -2.6 | - && - |
| gene:GYO_4396 | 2.4 | -1.0 | -1.7 | -1.5 | - && PF06271:RDD family |
| gene:GYO_2119 | -1.2 | 1.7 | -2.0 | -2.1 | - && PF00083:Sugar (and other) transporter |
| gene:GYO_4285 | -1.4 | 1.8 | -2.1 | -2.8 | transporter\|PF03459:TOBE domain\|PF07673:Protein of unknown function (DUF1602) |
| gene:GYO_4341 | -1.0 | 1.0 | -1.8 | -1.5 | - && PF09021:HutP |
| gene:GYO_4342 | -1.3 | 2.0 | 1.9 | 1.6 | - && PF00221:Aromatic amino acid lyase |
| gene:GYO_4343 | -1.2 | 2.1 | 2.5 | 2.3 | - && PF01175:Urocanase |

[a] $log_2$FoldChange
