# Supplementary material for: Disparate Effects of Two Clerodane Diterpenes of Giant Goldenrod (Solidago gigantea Ait.) on Bacillus spizizenii
Source: Int J Mol Sci. 2024 Jan 26;25(3):1531. doi: 10.3390/ijms25031531 (PMC10855248; doi:10.3390/ijms25031531)
Supplement: Supplementary file 1 [file ijms-25-01531-s001.zip › Table S3.pdf]

Table S3. KEGG pathways enrichment results of diterpene treatments.

	KEGG pathways	Sg3a-5h				Sg6-5h				Sg3a-1h				Sg6-1h			
		UP/DOWN		UP	DOWN	UP/DOWN		UP	DOWN	UP/DOWN		UP	DOWN	UP/DOWN		UP	DOWN
bst02010	ABC transporters	35[a]	36														12
bst02030	Bacterial chemotaxis				20	2	21		21				9				5
bst02040	Flagellar assembly	2	28		28	1	37		37				12				
bst00552	Teichoic acid biosynthesis			13													
bst00300	Lysine biosynthesis			9													
bst00230	Purine metabolism					10	34		34								
bst00240	Pyrimidine metabolism					3	23		23								
bst00640	Propanoate metabolism					9	15										
bst01120	Microbial metabolism in diverse environments					72	45	72									
bst01110	Biosynthesis of secondary metabolites					83	112					48		65	13	65	
bst00650	Butanoate metabolism					13	6	13									
bst00190	Oxidative phosphorylation					3	25		25								
bst03010	Ribosome								34								
bst01232	Nucleotide metabolism								29								
bst00670	One carbon pool by folate								9								
bst00500	Starch and sucrose metabolism							23									
bst00920	Sulfur metabolism							11									
bst00562	Inositol phosphate metabolism							9									
bst02060	Phosphotransferase system (PTS)							15									
bst00520	Amino sugar and nucleotide sugar metabolism							16									
bst00071	Fatty acid degradation							8				8					
bst04122	Sulfur relay system											7					
bst00740	Riboflavin metabolism											6					
bst01240	Biosynthesis of cofactors											29					
bst00130	Ubiquinone and other terpenoid-quinone biosynthesis											6		7	1	7	
bst01230	Biosynthesis of amino acids													32	5	32	
bst00220	Arginine biosynthesis							10								8	
bst00340	Histidine metabolism													6	1	6	
bst00380	Tryptophan metabolism															6	
bst00290	Valine, leucine and isoleucine biosynthesis															9	
bst00280	Valine, leucine and isoleucine degradation													7	1	7	
bst00561	Glycerolipid metabolism													4	4		4
bst00630	Glyoxylate and dicarboxylate metabolism													10	1	10	
bst01210	2-Oxocarboxylic acid metabolism							14								15	
bst00010	Glycolysis / Gluconeogenesis															10	
bst00400	Phenylalanine, tyrosine and tryptophan biosynthesis																5

[a]**The number of the genes up- or down-regulated in the significantly enriched pathways is marked in red and green, respectively.**
