# Supplementary figures and images for: Disparate Effects of Two Clerodane Diterpenes of Giant Goldenrod (Solidago gigantea Ait.) on Bacillus spizizenii
Source: Int J Mol Sci. 2024 Jan 26;25(3):1531. doi: 10.3390/ijms25031531 (PMC10855248; doi:10.3390/ijms25031531)

Sg3a-5h vs. control-5h

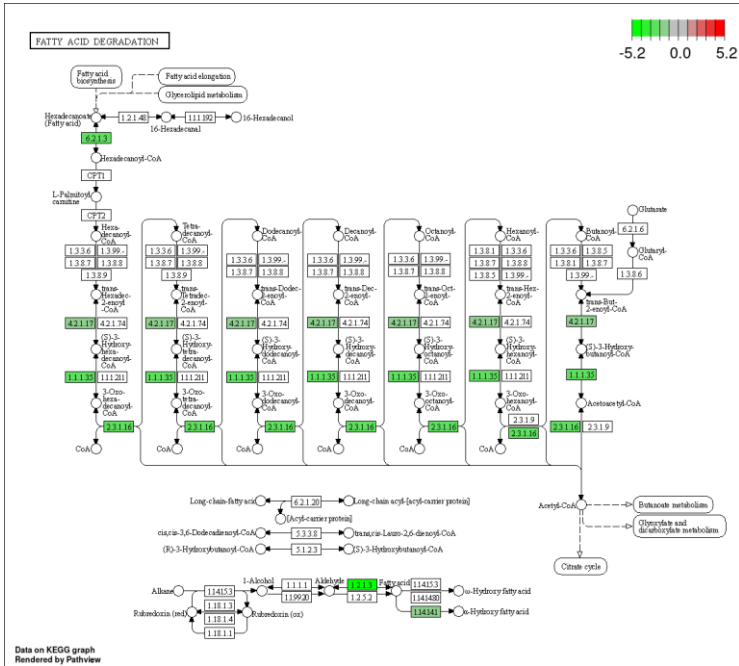

Sg6-5h vs. control-5h

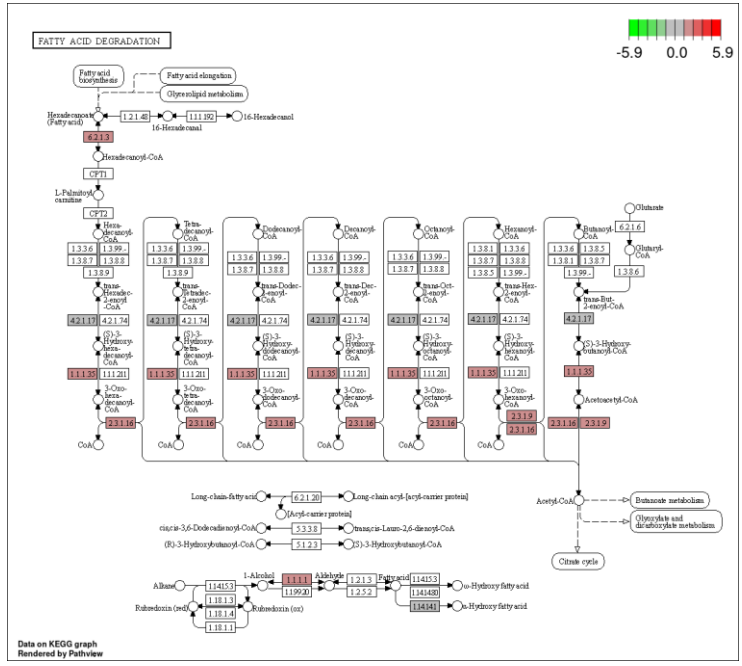

Sg3a-1h vs. control-1h

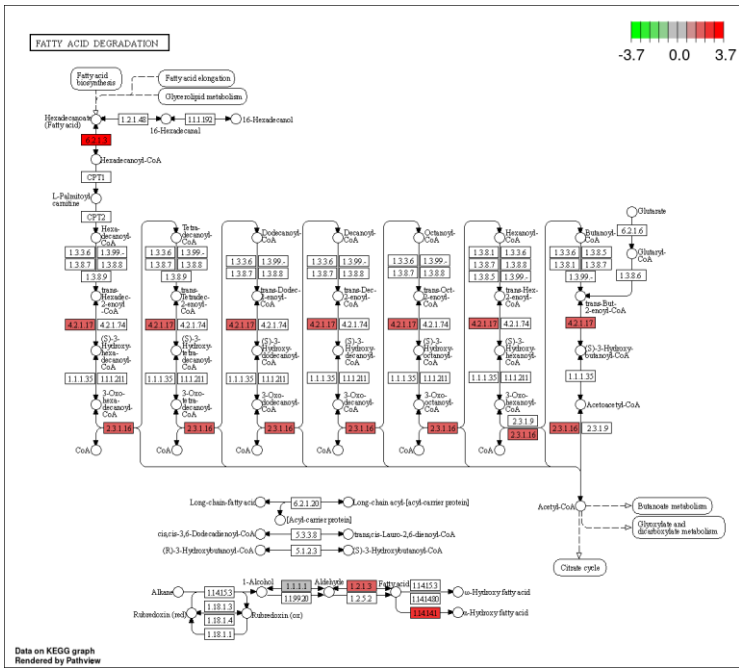

Sg6-1h vs. control-1h

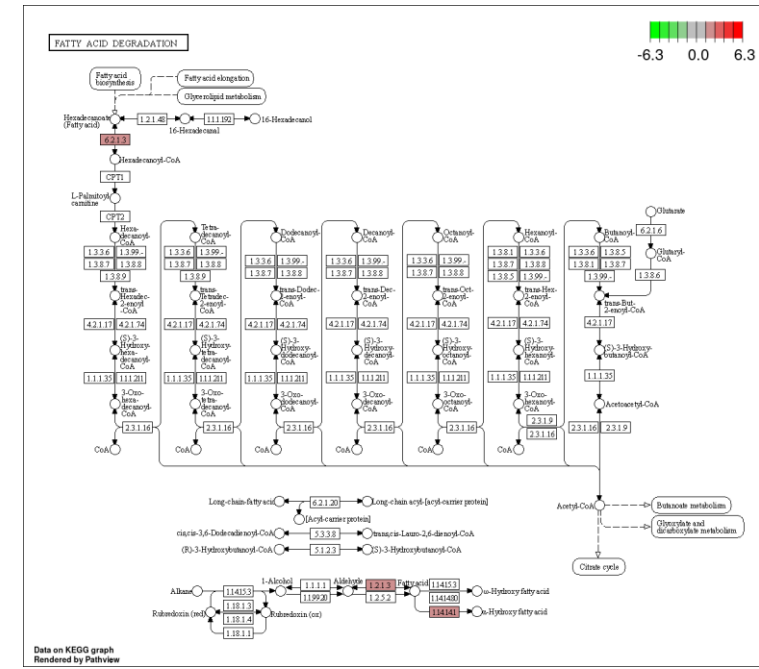

Supplement: Supplementary file 1 [file ijms-25-01531-s001.zip › Figure S3.pdf]
